# Studies on the laccase-mediated decolorization, kinetic, and microtoxicity of some synthetic azo dyes

**DOI:** 10.1186/s40201-016-0248-9

**Published:** 2016-05-13

**Authors:** Hamid Forootanfar, Shahla Rezaei, Hamed Zeinvand-Lorestani, Hamed Tahmasbi, Mehdi Mogharabi, Alieh Ameri, Mohammad Ali Faramarzi

**Affiliations:** Department of Pharmaceutical Biotechnology, Faculty of Pharmacy, Kerman University of Medical Sciences, Kerman, Iran; Department of Pharmaceutical Biotechnology, Faculty of Pharmacy and Biotechnology Research Center, Tehran University of Medical Sciences, P.O. Box 14155-6451, Tehran, 1417614411 Iran; Department of Pharmacology and Toxicology, Faculty of Pharmacy, Tehran University of Medical Sciences, P.O. Box 14155-6451, Tehran, 1417614411 Iran; Department of Medicinal Chemistry, Faculty of Pharmacy, Kerman University of Medical Sciences, Kerman, Iran

**Keywords:** Decolorization, Detoxification, Laccase, Hydroxybenzotriazole, Microtoxicity

## Abstract

**Background:**

Enzymatic elimination of synthetic dyes, one of the most environmentally hazardous chemicals, has gained a great interest during the two last decades. The present study was performed to evaluate the decolorization and detoxification potential of the purified laccase of *Paraconiothyrium variabile* in both non-assisted and hydroxybenzotriazole-aided form against six azo dyes.

**Results:**

The obtained results showed that Acid Orange 67, Disperse Yellow 79, Basic Yellow 28, Basic Red 18, Direct Yellow 107, and Direct Black 166 were decolorized up to 65.3, 53.3, 46.7, 40.7, 34, and 26.2 %, respectively, after 1 h treatment with laccase (0.5 U/mL). Addition of HBT up to 5 mM enhanced decolorization percent of all the investigated dyes. The results of kinetic study introduced the monoazo dye of Acid Orange 67 as the most suitable substrate for laccase with *K*_*m*_ of 0.49 mM and *V*_*max*_ of 189 mmol/min/mg. Evaluation the toxic effect of laccase-treated dye sample based on the growth inhibition of standard bacterial strains revealed decrease in toxicity of all applied dyes after treatment by laccase.

**Conclusions:**

Application of the *P. variabile* laccase as biocatalyst efficiently decreased the toxicity of all studied synthetic azo dyes.

## Background

Synthetic dyes are being increasingly applied in the textile, paper, cosmetics, pharmaceutical, leather dyeing, color photography, and food industries [1–4]. Azo dyes (contain the N = N group in their structure) identified as one of the most popular synthetic colorant agent because they could be easily and affordably synthesized and found to be stable [5–7]. However, the toxicity, mutagenicity, and carcinogenicity of synthetic azo dyes and/or their metabolites have been well documented [8–10]. Furthermore, the harmful effects of azo dyes on the germination and growth of many environmentally important plants have been well described [1, 10]. So, development of physicochemical [11, 12] and/or biological techniques for treatment of wastewater rich in such complex aromatic structures received great attention during the two last decades among which enzymatic removal of such pollutants is an economic and environmentally friendly procedure due to the low energy required and the minimal impact on ecosystems [13–15].

Laccases (EC 1.10.3.2), the multicopper-containing oxidases belonging to the superfamily of multicopper oxidase, are mainly produced by plants, fungi especially the white-rot basidiomycetes and some bacterial strains [16–18]. Laccases alone or in assistance with mediators have been found to catalyze the oxidation of a broad range of substrates such as phenol and its derivatives, benzenethiols, aromatic amines, and polycyclic aromatic hydrocarbons (PAHs) [19–21]. The mentioned feature launched these biocatalysts as the main tool in the xenobiotic removal studies [22, 23]. There are many reports on the application of the purified laccases or laccase-producing organisms for elimination of synthetic and natural dyes [24, 25]. For example, the ability of laccase from *Trametes versicolor* for decolorization of four dyes including Red FN-2BL, Red BWS, Remazol Blue RR and Blue 4BL was reported by Mendoza et al. [26]. In the study of Yan et al. [27] two azo dyes including reactive black and congo red decolorized by the crude laccase of *Trametes trogii* S0301.

In the present study the potential application of the purified laccase originated from a soil-isolated fungal strain [25] for decolorization and detoxification of 6 synthetic azo dyes was evaluated. Thereafter, the effects of factors including pH and temperature as well as enzyme and mediator concentration on decolorization percent were also demonstrated. Additionally, the energetic (ΔS, ΔH, and Ea) and kinetic (*K*_m_ and *V*_max_) parameters for laccase-mediated decolorization were determined.

## Methods

### Chemicals and enzyme

**Fig. 1:**
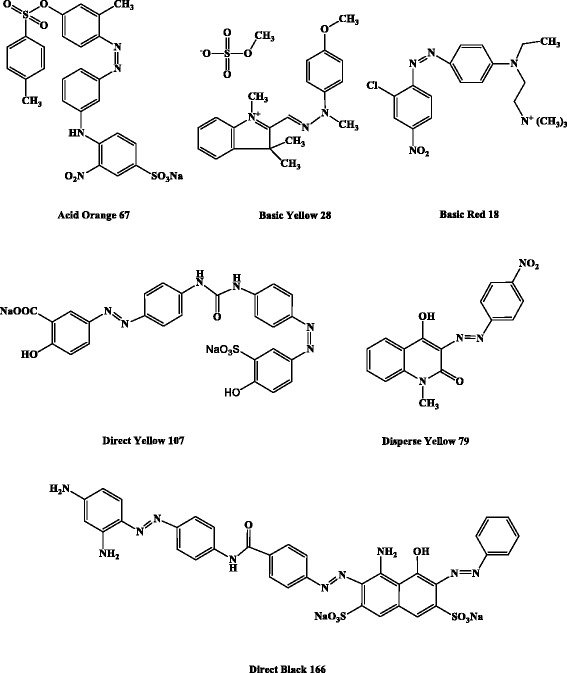
Chemical structures of azo dyes applied in the present study

The six applied azo dyes (Fig. 1 and Table 1) were supplied by Alvan Sabet Co. (Tehran, Iran). 2,2’-Azinobis-(3-ethylbenzothiazoline-6-sulfonate) (ABTS) and 1-hydroxybenzotriazole (HBT) were provided by Sigma-Aldrich (St. Louis, MO, USA) and Merck (Darmstadt, Germany), respectively. The laccase applied in the present study was purified as previously described [25].

**Table 1:** Kinetic and energetic parameters of the laccase on studied synthetic dyes

Dye name	Type	λ_max_ (nm)	*K*_m_ (mM)	*V*_max_ (mmol/min/mg)	ΔS (Kj/mol/K)	ΔH (kJ/mol)	Ea (kJ/mol)
Acid Orange 67	Monoazo	438	0.49	189	307	93	24
Basic Red 18	Monoazo	489	3.07	64	194	63	28
Basic Yellow 28	Monoazo	450	2.07	100	308	91	29
Disperse Yellow 79	Monoazo	474	2.02	104	189	58	30
Direct Yellow 107	Diazo	368	3.44	47	213	68	26
Direct Black 166	Triazo	602	4.25	33	199	65	31

### Laccase assay

The previously reported method of Forootanfar et al. [25] using ABTS was applied for measuring the oxidative activity of laccase. In brief, into the 0.5 mL freshly prepared enzyme substrate (ABTS 5 mM in citrate buffer 100 mM pH 5) enzyme sample (0.5 mL) was added and the reaction mixture was incubated at 37 °C and 120 rpm for 10 min. Afterwards, the absorbance was recorded at 420 nm (ε_420_ = 36,000 M^−1^ cm^−1^) by a Double Beam PC Scanning UV–vis spectrophotometer (UVD 2950, Labomed, Culver City, USA). The amount of enzyme which oxidize 1 μmol of ABTS per minute defined as one unit of laccase activity [28–31].

### Dye decolorization experiments

The previously described method of Ashrafi et al. [5] was used in order to assess the decolorization potential of the purified laccase. Firstly, dye solution was prepared by dissolving of each synthetic dye in citrate-phosphate buffer (100 mM, pH 5.0). Thereafter, the purified enzyme (final activity of 0.5 U/mL) was inserted into each dye solution and the reaction mixture was incubated at 35 °C and 50 rpm for 3 h. Subsequently, interval samples were taken each 30 min and the related absorbance (at the λ_max_ of each dye, Table 1) was measured and decolorization percent was calculated using the below equation

$$ \mathrm{Decolorization}\ \left(\%\right) = \left[{\mathrm{A}}_{\mathrm{i}}\hbox{--}\ {\mathrm{A}}_{\mathrm{t}}/\ {\mathrm{A}}_{\mathrm{i}}\right] \times 100 $$

where A_i_ and A_t_ are the initial absorbance and the absorbance after incubation time of the reaction mixture, respectively [32, 33]. In order to confirm about significant change in decolorization percentage the reaction mixture was further incubated overnight. To examine the abiotic decolorization (negative control) each dye solution was inserted by heat-inactivated laccase and the related decolorization percent was estimated as said above. Three independent experiments were conducted for each dye and means of decolorization percent was calculated.

### The influence of pH alteration on enzymatic decolorization

In order to investigate the influence of pH on laccase-mediated decolorization each dye solution was firstly prepared in 0.1 M citrate-phosphate buffer (pH 3–7) or Tris-HCl buffer (0.1 M for pH 8) and the purified laccase (0.5 U/mL) was consequently added to the reaction mixture which was then monitored for decolorization as previously described.

### The effect of temperature on laccase-mediated decolorization

After preparation of the reaction mixture including each studied dye and the purified laccase (0.5 U/mL) it was incubated at different temperatures (5–75 °C) and decolorization percent was then determined using the previously described procedure.

### Laccase activity influence on dye decolorization

The effect of enzyme concentration on decolorization process was evaluated by insertion of laccase (final activity range of 0.5–5 U/mL) into the freshly prepared dye solution (in 0.1 M citrate-phosphate buffer pH 5) and incubation at 35 °C and 50 rpm for 1 h followed by recording of the related absorbance and calculation of the decolorization percent.

### The effect of laccase mediator (HBT) on decolorization

After dissolving of each dye into citrate-phosphate buffer (0.1 M pH 5) HBT was also inserted (final concentrations of 1, 5, and 10 mM) and decolorization was initiated by addition of laccase (0.5 U/mL) into the reaction mixture. The amount of decolorization was then demonstrated as mentioned above.

### The kinetics and energetics of dye elimination

#### Decolorization kinetics

In order to acquire the kinetic parameters of decolorization the related velocity (V) was firstly obtained by performing dye decolorization at different concentrations (C) of each dye. The Michaelis-Menten curve was then drawn by plotting the obtained velocity against concentration. Consequently, Lineweaver-Burk transformation was applied in order to obtain the kinetic constant (*K*_m_ and *V*_max_) of decolorization for each dye [5, 10].

### Decolorization thermodynamics

The related velocity of decolorization was initially determined at various temperature of 10–50 °C and applied for drawing of a curve by plotting the calculated velocity against initial dye concentration. The apparent first-order rate constant (K) was then calculated based on the slope of the straight plot achieved for each dye and employed to drain the linearized Arrhenius curve by plotting the ln (K) versus 1/T (×10^3^ K^−1^) where T is the absolute temperature (K). The slope of Arrhenius plot indicates -E_a_/R in which Ea is the activation energy and R is the gas constant (8.3145 J /mol/K). Thereafter, Van’t Hoff curve was drained by plotting the ln (K_eq_) against 1/T (×10^3^ K^−1^) and applied for determination of the entropy (ΔS) and enthalpy (ΔH) of decolorization reaction. K_eq_ is the apparent equilibrium constant which was determined from difference of initial and remained dye concentration at equilibrium state, when the decolorization percentage become constant and no decolorization was occurred with passing the time [34]. Subsequently, the Gibbs free energy (ΔG) was also calculated using the equation of ΔG = ΔH– TΔS.

### Dye toxicity

A modified microtoxicity assay procedure using six standard bacterial strain (three Gram-negative and three Gram-positive species, Table 2) was employed to determine the toxicity of each studied dye before and after treatment by the purified laccase. In brief, a proper dilution of each bacterial suspension (OD_600_ 0.2) in Muller-Hinton broth medium was firstly prepared and successively inserted by each dye or its related laccase-treated solution and incubated at 37 °C for 10 h. Afterwards, interval samples were taken every 2 h and analyzed for changes in the OD_600_ compared to that of the negative control (cultivated bacterial strain in the absence of any dye). The equation of [(1 - OD_600S_/ OD_600C_) × 100] was then utilized for determination of growth inhibition percent (GI%), where OD_600S_ and OD_600C_ are OD_600_ of sample and OD_600_ of control, respectively.

**Table 2:** Growth inhibition percent (GI%) of untreated and laccase-treated dyes against six bacterial strains. Values are averages of three replicates ± standard deviation

Bacterial strains												
Dye names	*E. coli*		*P. aeroginosa*		*S. typhi*		*B. subtilis*		*S. aureus*		*M. luteus*	
	U^a^	T^b^	U	T	U	T	U	T	U	T	U	T
Acid Orange 67	28 ± 1.1	19 ± 0.5^*^	21 ± 0.4	13 ± 0.6^*^	24 ± 0.8	16 ± 0.4^*^	37 ± 1.1	28 ± 1.0^*^	33 ± 1.8	20 ± 0.8^*^	42 ± 2.3	31 ± 0.6^*^
Basic Red 18	25 ± 1.1	14 ± 0.4^*^	18 ± 1.2	8 ± 0.3^*^	23 ± 1.2	11 ± 0.7^*^	34 ± 1.5	21 ± 0.5^*^	30 ± 1.1	19 ± 0.6^*^	37 ± 0.8	23 ± 0.7^*^
Basic Yellow 28	32 ± 1.8	20 ± 0.4^*^	26 ± 1.4	16 ± 0.3^*^	29 ± 1.3	17 ± 0.6^*^	41 ± 1.9	30 ± 0.7^*^	36 ± 0.7	28 ± 0.6^*^	49 ± 1.2	35 ± 1.1^*^
Disperse Yellow 79	21 ± 0.9	9 ± 0.4^*^	14 ± 0.7	5 ± 0.3^*^	18 ± 0.3	7 ± 0.4^*^	27 ± 0.4	12 ± 0.2^*^	23 ± 0.9	9 ± 0.3^*^	30 ± 1.1	16 ± 0.5^*^
Direct Yellow 107	46 ± 1.4	34 ± 0.4^*^	38 ± 1.0	29 ± 0.3^*^	41 ± 0.8	31 ± 0.8^*^	57 ± 0.6	44 ± 0.6^*^	49 ± 0.9	41 ± 0.9^*^	62 ± 0.7	48 ± 0.7^*^
Direct Black 166	51 ± 1.8	37 ± 0.7^*^	43 ± 1.2	30 ± 0.6^*^	47 ± 1.9	31 ± 0.8^*^	61 ± 2.2	50 ± 0.9^*^	56 ± 2.1	45 ± 0.8^*^	68 ± 2.9	54 ± 1.0^*^

^a^GI% in presence of untreated dye solution; ^b^GI% in presence of laccase-treated dye solution; ^*^Significancy was determined using independent sample *t*-test (*p*-value < 0.05)

### Statistical analysis

Three replicates of each above mentioned experiment was performed and mean ± standard deviation of each value was reported. In order to calculate the statistical significance (probability values less than 0.05) between mean values the independent sample *t*-test and one-way analysis of variance (ANOVA) with Dunnett’s T3 post hoc test were employed using the SPSS software (version 15.0, SPSS Inc).

## Results and discussion

### Decolorization studies

**Fig. 2:**
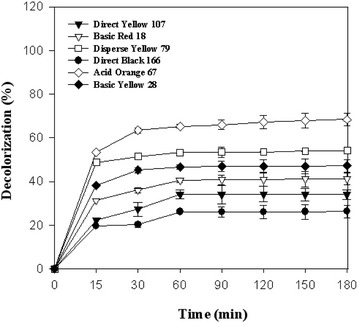
Laccase mediated (0.5 U/mL) decolorization profiles of applied dyes during 180 min of incubation at 35 °C

As presented in the time course of decolorization of six synthetic azo dyes (Fig. 2), laccase was able to efficiently decolorize all studied dyes within 3 h incubation. Acid Orange 67, Disperse Yellow 79, Basic Yellow 28, Basic Red 18, Direct Yellow 107, and Direct Black 166 were respectively decolorized up to 65.3, 53.3, 46.7, 40.7, 34, and 26.2 % after 1 h treatment with laccase. Elongation of the reaction time to 24 h didn’t significantly enhance decolorization percent (data not shown). Furthermore, for abiotic control (the reaction mixture without enzyme) no decolorization was observed indicating the catalytic role of laccase in the decolorization procedure.

Application of laccases and laccase mediated system (LMS) to decolorize mutagenic and carcinogenic synthetic colorants has been received great interest during two last decades [17, 35]. Literature review revealed that laccase assisted decolorization pattern even in the case of one special dye could vary depends on the origin of laccases [1, 10]. There are many reports about decolorization ability of laccase and laccase mediated systems toward a broad range of synthetic dyes including diazo dyes, anthraquinone dyes, triphenylmethane dyes, and textile colorants [2, 5, 24]. The obtained results of the present study revealed the potential application of laccase for decolorization of six synthetic azo dyes (Fig. 2). Literature review introduced azo dyes as recalcitrant dyes using both the enzymatic and physicochemical procedures [1, 13]. In general, resistance of azo colorants to laccase-catalyzed oxidation enhanced by increasing the number of azo group [36, 37]. For example, Ashrafi et al. [5] achieved the lowest and highest decolorization percent for Direct Blue 71 (a triazo dye, 30 %) and Acid Red 18 (a monoazo dye, 97 %), respectively, after 15 min treatment using the secreted laccase of *P. varibile*. In the study of Zeng et al. [38] it was found that the laccase originated by *T. trogii* SYBC-LZ didn’t decolorize Acid Red 1 (a monoazo dye) and Reactive Black 5 (a diazo dye) while the highest decolorization percent (80.4 %) achieved for the anthraquinonic dye of Remazol Brilliant Blue R (RBBR). Grassi et al. [8] demonstrated that only 7 % of Fast Blue RR (an azo dye) was removed after 1 h incubation in the presence of the purified laccase of *T. trogii* while more than 80 % of anthraquinonic dye of RBBR and Indigo Carmine was eliminated at the same time.

### The influence of pH and temperature on laccase-catalyzed decolorization

**Fig. 3:**
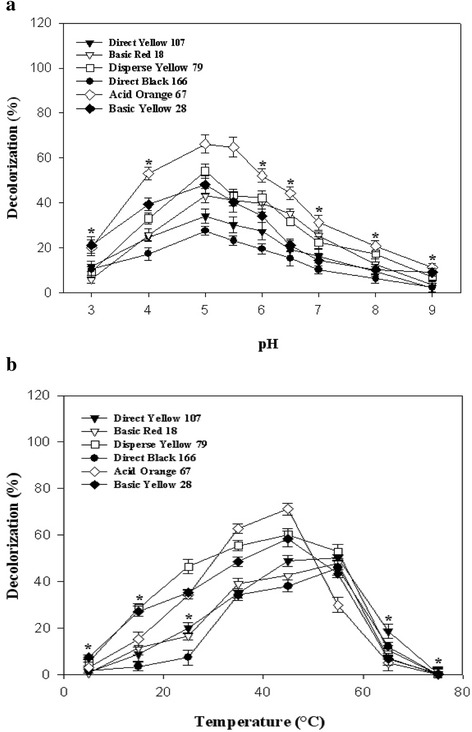
Effect of various (**a**) pH, and (**b**) temperatures on decolorization of azo dyes using the purified laccase (0.5 U/mL) after 60 min of incubation. Obtained means were analyzed by ANOVA with Dunnett’s T3 post hoc test (*, *p*-value <0.05)

The results of pH alteration on decolorization of investigated dyes were illustrated in Fig. 3a. Maximum decolorization of all dyes occurred at the optimum pH of the applied laccase (5) which previously reported by Forootanfar et al. [25]. Alteration from the optimum pH of laccase negatively affected decolorization percent of all synthetic dyes. In the study of Ashrafi et al. [5] and Mirzadeh et al. [10] laccase mediated decolorization of all applied dyes was maximally achieved at acidic pH of 5. It was demonstrated that most of laccases of fungal origins maximally work at acidic pH and the enzyme activity at higher pH is decreased due to binding of hydroxide anion to the T2/T3 coppers of laccase and as a result interrupting with the internal electron transfer from T1 to T2/T3 centers [39].

Decolorization percent of all studied dyes was gradually increased by enhancing the reaction temperature and maximum decolorization of Disperse Yellow 79 (60 %), Acid Orange 67 (71.3 %), and Basic Yellow 28 (58.4 %) was observed at the temperature of 45 °C (Fig. 3b). Basic Red 18 (48 %), Direct Yellow 107 (50.2 %), and Direct Black 166 (46 %) were maximally decolorized at 55 °C (Fig. 3b). The amount of decolorization of all studied dyes was dropped below 20 % (*p*-value < 0.05) by elevating of temperature to 70 °C which was in agreement with the acquired results of Ashrafi et al. [5] who observed that maximum dye decolorization occurred between the temperatures of 40–60 °C. In general, most of the fungal derived laccases optimally work at the temperature range of 50–70 °C [39].

### The effect of laccase activity on decolorization

**Fig. 4:**
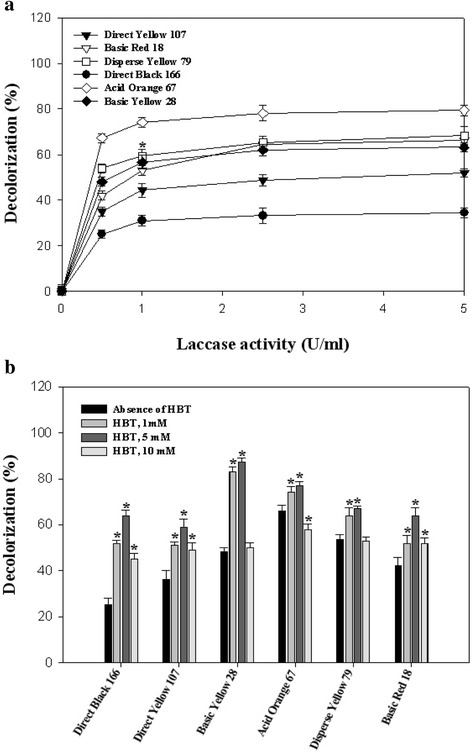
Influence of (**a**) laccase activity and (**b**) HBT concentration on decolorization of investigated dyes after 60 min of incubation at 35 °C. Significant values (*, *p*-value <0.05) were achieved after ANOVA analysis with Dunnett’s T3 post hoc test

As represented in Fig. 4a, the decolorization percent of all studied dyes (except for Acid Orange 67) was significantly enhanced (*p*-value < 0.05) after increasing of laccase activity from 0.5 U/mL to 1 U/mL. Maalej-Kammoun et al. [6] determined decolorization percent of 80 % for laccase-catalyzed (1 U/mL) elimination of Malachite green within 2 h of initiation of the reaction. In the study of Ashrafi et al. [5] it was showed that increasing of laccase activity from 0.025 to 0.1 U/mL significantly increased decolorization percent of all thirteen studied synthetic dyes.

### The influence of HBT on laccase mediated decolorization

As represented in Fig. 4b, decolorization percent of all synthetic dyes was positively affected by increasing of HBT concentration up to 5 mM and decreased by enhancing the HBT concentration more than 5 mM.

Treatment of hazardous organic pollutants and synthetic dyes using laccase-mediated systems, especially in the case of laccases with low redox potential and recalcitrant pollutants, has been well documented [22]. For example, Ostadhadi-Dehkordi et al. [40] investigated on the mediating activity of the phenol derivatives of vanillic acid (VA) and 2,6-dimethoxyphenol (DMP) and the non-phenolic mediators (ABTS and HBT), among which HBT was the most efficient one increased the removal percent of four investigated benzodiazepines (one of the most prescribed pharmaceuticals). Grassi et al. [8] evaluated the effect of synthetic (HBT) and several natural laccase mediators including ρ-hydroxybenzoic acid (HBA), tyrosine, vanillin, vanillic acid, anisaldehyde, and ferulic acid on decolorization of Azure B, Xylidine, and Gentian Violet in the presence of *T. trogii*’s laccase. They introduced HBT as the best mediator for maximum decolorization [8]. Laccase-HBT system is generally more effective compared to unaided laccase due to stronger oxidative activity of free-radical HBT species. Furthermore, high redox potential of HBT (1084 mV) together with the stabilizing activity of HBT on laccases makes it suitable for elimination of recalcitrant colorants using oxidative activity of laccases [22, 40]. However, depend on the source of laccases applied in the elimination studies, there is a critical concentration above which the assisting activity of this non-phenolic laccase mediator is negatively affected by the destructive effect of produced HBT radical on enzyme structure [22]. So, literature review revealed many reports on determination of optimum concentration of HBT to get maximum decolorization using laccase-HBT system [5, 40].

### Kinetics and energetic studies

Based on the kinetic parameters of the laccase toward studied dyes (Tabale 1) the lowest and the highest *K*_*m*_ value belonged to the monoazo dye of Acid Orange 67 (0.49 mM) and triazo dye of Direct Black 166 (4.25 mM), respectively. Unsuitability of Direct Black 166 as a laccase substrate is evident from the high value of the calculated *K*_*m*_ of this colorant (4.25 mM) and its related maximum velocity (33 mmol/min/mg). The resistance of azo dyes to laccase-catalyzed oxidation compared to other classes of colorants has been well documented. Grassi et al. [8] reported the lowest decolorization percent for the azo dye of Fast Blue RR using the purified laccase of *T. trogii* B6J among tested synthetic dyes. Generally, presence of electron withdrawing groups such as –SO_3_H, −SO_2_NH_2_ together with the high molecular weight decreases the ability of laccases for elimination of azo dyes [1]. Application of laccases harboring higher redox potential such as bacterial laccases and addition of synthetic or natural redox mediators are two main solutions to overcome such problem [1]. As an example, the recombinant bacterial CotA-laccase from *B. subtilis* was able to decolorize Direct Blue 1 (a diazo dye), Reactive Black 5 (a diazo dye), and Sudan Orange G (a monoazo dye) by 88, 85 and 98 %, respectively at alkaline pH and in the absence of redox mediators [9].

The obtained results of energetic assessment for laccase-mediated decolorization (Table 1) indicated the significant influence of temperature on decolorization of all investigated synthetic dyes as decolorization rate increased from 10 °C to 50 °C. The activation energies (calculated from the slope of Arrhenius plot) together with the estimated values for ΔH and ΔS were also represented in Table 1. Considering the negative amount of slope obtained for van’t Hoff plot or the positive signs of ΔH (Table 1) it was demonstrated that the decolorization reactions are endothermic.

### Microtoxicity study

The results of microtoxicity study were summarized in Table 2. The highest GI% belonged to the Direct Black 166 (a triazo dye) suppressing *M. luteus*, *S. aureus*, *B. subtilis*, *E. coli*, *P. aeruginosa*, and *S. typhi* by 68, 56, 61, 51, 43, and 47 %, respectively, when exposed to each bacterial strain for 10 h. On the other hand, the monoazoic dye of Disperse Yellow 79 represented GI% of 30, 23, 27, 21, 14, and 18 % for *M. luteus*, *S. aureus*, *B. subtilis*, *E. coli*, *P. aeruginosa*, and *S. typhi*, respectively (Table 2) which was the lowest observed value. The GI% of all synthetic azo dyes was significantly reduced following laccase treatment as presented in Table 2.

In order to evaluate the probable toxicity of colorants and their metabolite(s) after physicochemical or biological treatment many biological assays such as inhibition the growth of bacterial, yeast, and mammalian cell lines as well as phytotoxicity evaluations on plant seeds were developed [5, 9, 41, 42]. Like the acquired results reported by Ashrafi et al. [5] the attained results of the present investigation showed that the toxicity of all applied dyes was significantly decreased after addition of laccase to dye solution. Younes et al. [13] found that the GI% of *Bacillus megaterium* was enhanced from 2 to 99 % after incubation of malachite green in the presence of purified laccase originated from *S. thermophilum*. Maalej-Kammoun et al. [6] reported that decolorization of malachite green resulted in the removal of its toxicity against *Phanerochaete chrysosporium*. However, Mendes et al. [14] found lower toxicity for intact azo dyes of Direct Black 38, Direct Red 80, Reactive Black 5, Reactive Yellow 145, Acid Black 194, and Acid Red 266 compared to that of laccase-treated sample in the presence of the yeast *S. cerevisiae*.

## Conclusion

The potential activity of the purified laccase of *P. variabile* for decolorization of 6 azo dyes in the presence and absence of HBT as the laccase mediator was evaluated. All the synthetic dyes were decolorized using non-assisted laccase. In addition, decolorization percent of all applied colorants improved in the presence of HBT (up to 5 mM) as laccase mediator. Evaluation the toxicity of laccase-treated dye samples assisted by growth inhibition percentage (GI%) of standard bacterial strains revealed significant decrease in the toxicity of all studied dyes after treatment by laccase.
